# A Challenging Twist in Pulmonary Renal Syndrome

**DOI:** 10.1155/2014/516362

**Published:** 2014-11-27

**Authors:** Rajaie Namas, Bernard Rubin, Wamidh Adwar, Alireza Meysami

**Affiliations:** ^1^Department of Internal Medicine, Division of Rheumatology, Henry Ford Hospital, Wayne State University, Detroit, MI 48202, USA; ^2^Department of Internal Medicine, Division of Rheumatology, University of Michigan, 1150 W. Medical Center Drive, SPC 5680, Ann Arbor, MI 48109, USA; ^3^Department of Pathology, Henry Ford Hospital, Wayne State University, Detroit, MI 48202, USA

## Abstract

*Case*. We report a rare case of hydralazine-induced anti-neutrophil cytoplasmic antibody-associated vasculitis. A 75-year-old African American woman with history of high blood pressure on hydralazine for 3 years presented with acute onset of shortness of breath and hemoptysis. Lab workup revealed a severe normocytic anemia and a serum creatinine of 5.09 mg/dL (baseline 0.9). Bronchoscopy demonstrated active pulmonary hemorrhage. A urine sample revealed red cell casts and a renal biopsy demonstrated pauci-immune, focally necrotizing glomerulonephritis with small crescents consistent with possible anti-neutrophil cytoplasmic antibody-positive renal vasculitis. Serologies showed high-titer MPO-ANCA and high-titer anti-histone antibodies. She was treated with intravenous steroids and subsequently with immunosuppression after cessation of hydralazine. The patient was subsequently discharged from hospital after a rapid clinical improvement. *Conclusion*. Hydralazine-induced anti-neutrophil cytoplasmic antibody-positive renal vasculitis is a rare adverse effect and can present as a severe vasculitic syndrome with multiple organ involvement. Features of this association include the presence of high titer of anti-myeloperoxidase anti-neutrophil cytoplasmic antibody with multiantigenicity, positive anti-histone antibodies, and the lack of immunoglobulin and complement deposition. Prompt cessation of hydralazine may be sufficient to reverse disease activity but immunosuppression may be needed.

## 1. Case Report

A 75-year-old African American female with history of hypertension and Type 2 DM presented to the emergency room with acute onset of shortness of breath and hemoptysis of 2-day duration. She denied fever, chest pain, recent hospitalizations, new medications, or travel history. Medications include gabapentin 100 mg at bedtime, hydralazine 50 mg every 8 hours for the past 3 years, and simvastatin 20 mg at bedtime. Four months ago, she was evaluated in the rheumatology clinic for multiple joint pains and swelling including hands and ankles. She denied fever, dry cough, dyspnea, chest pain, or hemoptysis. She also denied patchy alopecia, photosensitivity, mucosal or nasal ulcers, skin rash, or inflammatory eye disease. She was lost to followup before lab was done.

**Figure 1:**
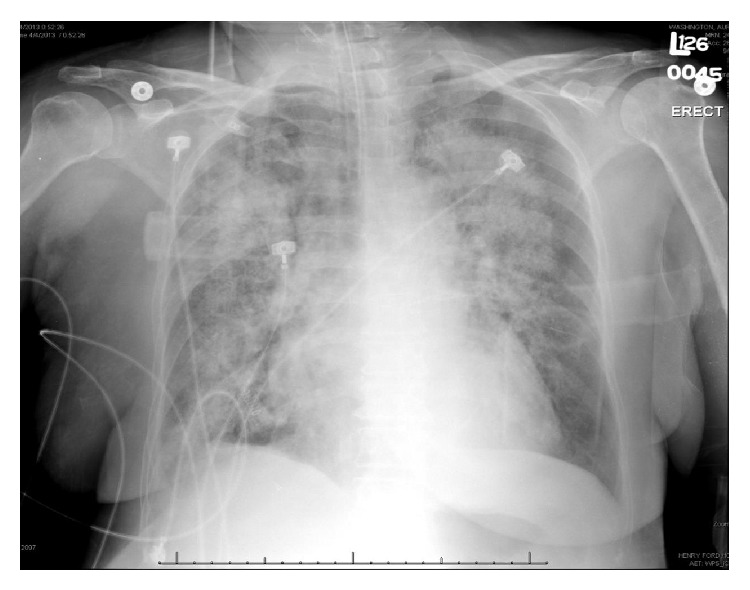
Chest X-ray revealing bilateral perihilar air space opacity. Tiny right pleural effusion with thickening or fluid within the fissures.

Physical exam in the emergency room revealed an intubated patient on mechanical ventilation who was hypertensive, tachypneic, and afebrile. Lung exam demonstrated diffuse coarse rales bilaterally. The rest of the physical exam was unremarkable. Laboratory findings on admission revealed ESR: 98 mm/hr, CRP: 7 mg/L, hemoglobin: 4.9 g/dL (baseline 11 g/dL), leukocytosis, lymphopenia, and serum creatinine of 5.09 mg/dL (baseline 0.9 mg/dL). Urine analysis demonstrated 3+ blood, 2+ protein, and red blood cell casts. Chest X-ray revealed bilateral perihilar air space opacity and a tiny right pleural effusion with thickening or fluid within the fissures (Figure 1).

During the hospital stay she received 2 units of blood and a bronchoscopy was consistent with active pulmonary hemorrhage. She was treated with 1 gram of methyl prednisolone IV; she was then switched to 1 mg/kg for possible pulmonary renal syndrome. Extensive evaluation with cultures (sputum, bronchoalveolar lavage, blood, and urine) and serologies for atypical infections including viral and fungal diseases failed to demonstrate an infectious etiology. Further workup demonstrated ANA of 1 : 320 (homogenous pattern), P-ANCA positive with anti-MPO of 52 and anti-PR3 of 28, and a strongly positive histone antibody (2.6, normal value 0–0.9). Anti-Smith (anti-Sm) antibody test was negative, and C3 and C4 complement levels were normal. Transthoracic echocardiography revealed severely elevated pulmonary artery systolic pressure (61 mmHg) with an ejection fraction of 55–60%. Ultrasound of the kidneys was normal.

**Figure 2:**
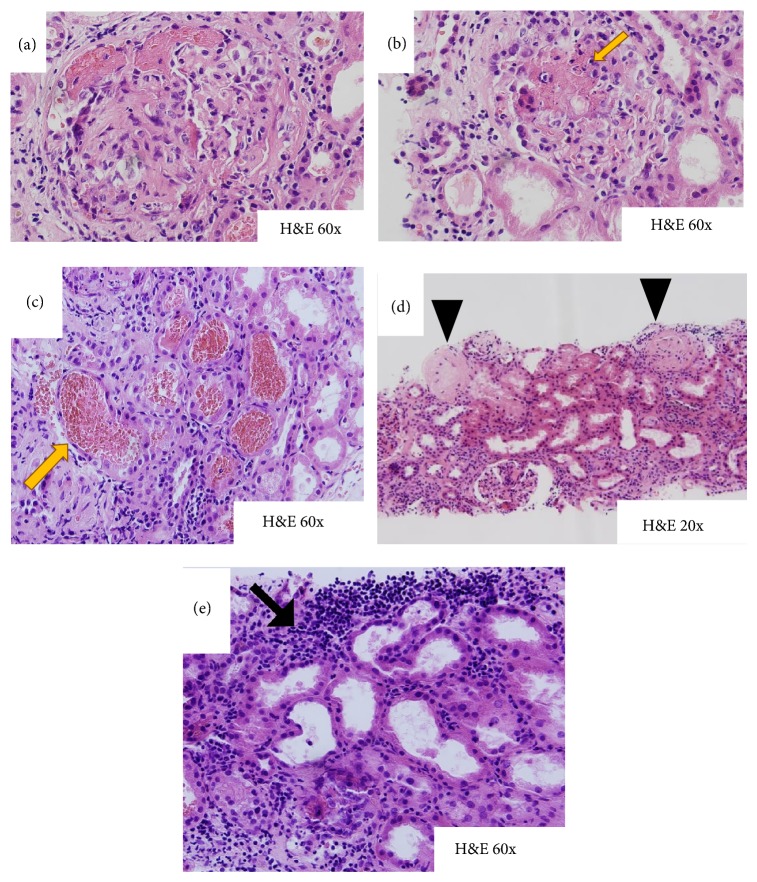
(a) Necrotizing glomerulonephritis with fibrocellular crescents (66%). (b) Global glomerulosclerosis (33%), focal infiltration of polymorphonuclear cells within the glomerulus (arrow). (c) Tubular hemorrhage (arrow). (d) Sclerosed glomeruli (arrow). (e) Interstitial inflammation lymphocytes, plasma cells, and polymorphonuclear cells (arrow).

**Figure 3:**
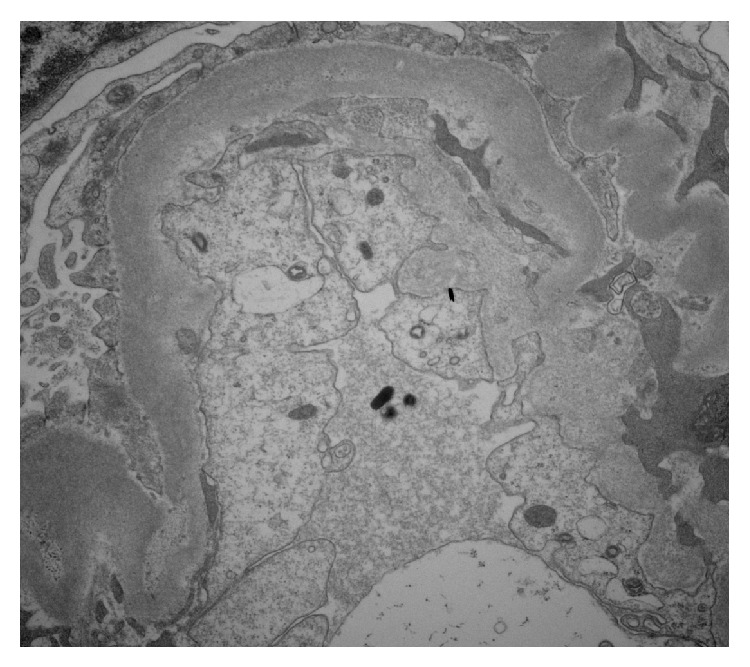
No evidence of electron dense, immune complex deposits by electron microscopy.

Due to her critical condition a kidney biopsy was not obtained on the day of admission but on day 5 her condition stabilized and a kidney biopsy was obtained which demonstrated necrotizing glomerulonephritis with fibrocellular crescents (66%) (Figure 2(a)), global glomerulosclerosis (33%), focal infiltration of polymorphonuclear cells within the glomerulus (arrow) (Figure 2(b)), tubular hemorrhage (Figure 2(c)), sclerosed glomeruli (Figure 2(d)), and interstitial inflammation with infiltration of lymphocytes, plasma cells, and polymorphonuclear cells (Figure 2(e)) on H&E stain. Direct immunofluorescence microscopy revealed peripheral granular positive staining, +1 of IgG, IgM, and C3, peripheral and mesangial positive staining, 1+ of IgA, and negative staining of fibrinogen, C1 and C4. There was peripheral granular positive staining, 1+ of kappa and lambda light chains, and nonspecific positive staining of albumin. Linear positivity was not seen with IgG or C3. Immunofluorescence studies and electron microscopy (Figure 3) were negative for immune complex deposition, consistent with pauci-immune glomerulonephritis.

A diagnosis of hydralazine-induced anti-neutrophil cytoplasmic antibody-positive renal vasculitis presenting with a pulmonary renal syndrome was established. To assess the probability that hydralazine caused the adverse event in our patient, we used the Naranjo adverse drug reaction probability scale patient's score which indicated that the association of hydralazine to pulmonary-renal syndrome was probable. Hydralazine was stopped and she was started on 750 mg of cyclophosphamide IV every 2 weeks and then was switched to oral cyclophosphamide (100 mg/day). Plasmapheresis was initiated on admission and she received a total of 7 cycles over 14 days of 60 mL/kg per session using 3% to 5% albumin as a replacement solution. The volumes were calculated using the estimated plasma volume (in liters) = 0.07 × wt (kg) × (1 − hematocrit). She was extubated on the 3rd day of admission. She received hemodialysis 3 times per week. Repeating chest X-ray and bronchoscopy 2 weeks later did not reveal any signs of pulmonary hemorrhage.

The patient was discharged on tapering dose of oral steroids, and an oral dose of 2 mg per kg of cyclophosphamide (100 mg/day) daily for 6 months with a cumulative dose was 21600 calculated based on the weight (weight (kg) × 2 mg) × 180 (days). One month later, she was asymptomatic, and the microscopic hematuria had resolved. Prednisone was weaned off over 3 months. One year later MPO-ANCA normalized, but the ANA remained positive.

## 2. Discussion

This case describes a patient with hydralazine-induced ANCA who presented with pauci-immune glomerulonephritis and the simultaneous presence of multiple autoantibodies (ANAs, anti-histone antibodies, and P-ANCA positive with anti-MPO and anti-PR3).

Hydralazine is a direct-acting smooth muscle vasodilator that is often used in the treatment of hypertension. Anti-neutrophil cytoplasmic antibody-associated vasculitis has been associated with many drugs such as allopurinol, sulfasalazine, and propylthiouracil and is a relatively rare side effect of hydralazine. The etiology of ANCA-associated vasculitis (AAV) is not always clear and this association is less well recognized compared to drug-induced lupus, which is well documented in the literature [1]. The diagnosis and management of patients may be challenging because of its relative infrequency. The incidence of hydralazine-induced vasculitis is dose dependent. It is reported to be 5.4% in patients on 100 mg/d of hydralazine versus 10.4% with 200 mg of daily dosing for ≥3 years duration, particularly in individuals who are slow acetylators. The key to the diagnosis is resolution of symptoms with discontinuation of the drug [2].

The spectrum of AAV can range from cutaneous rashes and petechiae or single organ involvement to fatal multiorgan involvement with death commonly from massive pulmonary hemorrhage. A latency of several years can occur before the development of vasculitis with a variable delay in the full clinical manifestations [3] therefore posing a challenge for clinicians to achieve a clear diagnosis and treatment strategy. The presence of high titers of P-ANCA and anti-MPO with multiantigenicity, the positive anti-histone antibodies, and the lack of immunoglobulin and complement deposition histopathologically are features that have been described with drug-induced ANCA vasculitis [3, 4] rather than with drug-induced lupus or with primary vasculitis.

Risk factors that have been identified as predisposing factors for hydralazine-induced AAV include a cumulative dose of more than 100 grams, female sex, and a history of thyroid disease [5] as seen in our patient. Other risk factors include the human leukocyte antigen (HLA)-DR4 genotype, slow hepatic acetylation, and the null gene for C4 [6].

The mechanism for hydralazine-induced AAV is not fully understood but might be multifactorial. Hypotheses of immune system activation by drug metabolites with autoimmunity towards neutrophil proteins (including elastase and lactoferrin) and upregulation of ANCA antigens have been suggested [6]. The hydralazine dosage ranged from 50 to 300 mg per day, and the treatment duration varied from 0.73 to 120 months. Almost all the patients in the literature were positive for MPO antibodies and all of the patients in whom anti-histone antibodies were checked had positive results.

## 3. Conclusion

The diagnosis of drug-induced lupus and drug-induced vasculitis is challenging to the physician. In the former, characteristic symptoms include arthralgia, myalgia, fever, and serositis that improve after discontinuation, whereas the latter is predominantly an anti-neutrophil cytoplasmic antibody (ANCA) positive small vessel vasculitis involving the kidneys, skin, and lungs that resembles idiopathic ANCA-associated vasculitides, such as granulomatosis with polyangiitis and microscopic polyangiitis. Discontinuation of the offending agent must be the first intervention and rechallenging using the same medication after resolution of the symptoms should be avoided in addition to avoiding similar drug classes.

## Abbreviations

AAV: ANCA-associated vasculitis
H&E: Hematoxylin and eosin stain.
